# Systemic inflammatory response index: a potential mediator between substance use disorder and sleep disturbance

**DOI:** 10.3389/fphar.2026.1964417

**Published:** 2026-10-20

**Authors:** Longtao Yang, Yihui Tang, Xuemei Wang, Xiang Chen, Qinyu Zou, Yule Zeng, Qitong Chen, Jun Liu

**Affiliations:** 1 Department of Radiology, The Second Xiangya Hospital of Central South University, Changsha, China; 2 The Second Xiangya Hospital, Central South University, Changsha, China; 3 Experimental Center, Guizhou Center for Disease Control and Prevention, Guiyang, China; 4 School of Medical Imaging, Changsha Medical University, Changsha, China; 5 Department of General Surgery, The Second Xiangya Hospital of Central South University, Changsha, China; 6 Clinical Research Center for Breast Disease in Hunan Province, Changsha, China; 7 Clinical Research Center for Medical Imaging in Hunan Province, Changsha, China; 8 Department of Radiology Quality Control Center in Hunan Province, Changsha, China

**Keywords:** immune inflammation, machine learning, sleep disturbance, substance use disorder, systemic inflammatory response index

## Abstract

**Study objectives:**

Substance use disorder (SUD) and sleep disturbance share a complex, bidirectional relationship. However, the role of the systemic inflammation response index (SIRI) in this relationship remains to be explored, which will be beneficial for detecting SUD-related sleep disturbances.

**Methods:**

Utilizing diverse samples from the National Health and Nutrition Examination Survey (NHANES) (n = 2,533) as well as self-collected clinical data (n = 88), we explored the associations among SUD, SIRI, and sleep disturbance. In NHANES dataset, weighted logistic regression and multiple machine learning (ML) models were employed to assess the relationship between SIRI and sleep disturbance, as well as SIRI importance in SUD group. Mediation analysis examined the role of SIRI in mediating the SUD-sleep disturbance association. During clinical confirmation, a two-sample t-test was used to discover differences in SIRI between opioid use disorder (OUD) and healthy controls (HC). Correlation of SIRI and Pittsburgh Sleep Quality Index (PSQI) was performed by partial correlation within OUD group.

**Results:**

The analyses on NHANES datasets uncovered significant “SUD–SIRI–sleep disturbance” relationships, and SIRI ranked first in diagnosing sleep status of SUD subjects. Through validation via clinical datasets, OUD individuals also had higher SIRI levels and sleep disturbance risk than HC.

**Conclusion:**

Our findings suggest that SIRI statistically mediates the relationship between SUD and sleep disturbance in a cross-sectional context, positioning it as a potential adjunctive monitoring indicator for SUD-related psychiatric management. These results offer preliminary correlational evidence for mitigating sleep disruption through anti-inflammatory approaches, warranting further longitudinal and experimental validation.

## Introduction

Sleep, a naturally recurring arousal state of the brain and body, is essential for regulating physiological processes (e.g., cognition, mood). Chronic poor sleep quality compromises impulse control, decision-making, and emotion regulation (Guarana et al., 2021), which are important in the occurrence and development of substance use disorder (SUD) (Verdejo-Garcia and Albein-Urios, 2021). In individuals with SUD, sleep disturbances are frequently observed, manifesting during periods of active use, withdrawal, and recovery (Adan et al., 2024; Bolling et al., 2025). Sleep disturbances increase vulnerability to SUD, and chronic substance misuse impairs sleep quantity and quality, which in turn augments the dependence cycle and drug relapse risk (Bichon et al., 2025; Shell et al., 2025). Therefore, the relationship between sleep disturbances and SUD is reciprocal, wherein disrupted sleep can be taken as both a consequence and contributor to SUD.

Substance addiction also interacts with immune homeostasis and evokes systemic inflammatory responses (Morcuende et al., 2021; Kogan et al., 2020; Cao et al., 2025). Rather than any one specific substance, higher overall substance use might be linked to greater peripheral inflammation (Nusslock et al., 2024). Compared with HC, SUD individuals exhibited significantly higher peripheral concentrations of pro-inflammatory cytokines (e.g., tumor necrosis factor-α (TNF-α), Interleukin-6/8 (IL-6/8), C-reactive protein (CRP) (Wei et al., 2020; Zhu et al., 2023). Collectively, across various stages involving ongoing addiction, acute withdrawal, and long-term abstinence, subjects with substance addiction can be characterized by concomitant chronic sleep disturbance and a sustained inflammatory state (Zhang et al., 2021; Yang et al., 2026). Besides, sleep disturbance was associated with increases in systemic inflammation markers (e.g., CRP, IL-6) (Irwin et al., 2016). Although the associations among SUD, inflammation, and sleep disorder are well-documented at the phenomenological level, how these three domains interact comprehensively, and whether a composite, routine-accessible inflammatory marker could reflect this interaction remains to be elucidated.

Given its derivation from routine hematological parameters including neutrophil count, lymphocyte count, and monocyte count, systemic inflammation response indicator (SIRI) offers a practical and cost-effective measurement for systemic inflammatory assessment (Hayama et al., 2025; Gao et al., 2025). Increased SIRI suggests a predominance of pro-inflammation (Islam et al., 2024). It is a composite hematological index reflecting the imbalance between innate immune activation and adaptive immune suppression. Its prognostic value has been validated across diverse clinical settings (Islam et al., 2024). For instance, in a large colorectal surgery cohort (n = 1,331), SIRI outperformed 11 other inflammatory composites in predicting postoperative complications, demonstrating its utility for preoperative risk stratification (P et al., 2026). Mechanistically, SIRI integrates three immune dimensions (neutrophils and monocytes for acute/chronical inflammation; and lymphocytes for impaired adaptive immune function) to capture a disrupted homeostatic state. While isolated cell fluctuations may indicate transient physiological stress, their ratio-based dysregulation signifies a breakdown of immune equilibrium. The multiplicative amplification enables SIRI to sensitively identify individuals at heightened risk of disease progression or poor therapeutic outcomes. Moreover, SIRI also quantifies the overall or local regulation of immune responses (Wu et al., 2023). SIRI level has been reported to be aberrant in psychiatric diseases (e.g., SUD, sleep disorder, depression) (Yang et al., 2025; Sehlikoğlu et al., 2024; Wang et al., 2025; Gong et al., 2025). For example, compared to controls, higher SIRI could be seen in methamphetamine and cannabis users (Sehlikoğlu et al., 2024). In view of its well accessibility and cost efficiency, we hypothesize that SIRI may serve as a supplementary biological indicator for identifying SUD individuals who exhibit a pro-inflammatory phenotype concomitant with severe sleep disturbances, a subgroup potentially more vulnerable to relapse and treatment resistance. Although standardized self-report scales (e.g., ISI, PSQI) are indispensable for evaluating perceived sleep quality in clinical care, they are inherently limited by recall bias, and crucially, they cannot capture the underlying systemic immuno-inflammatory state that is a key driver of the sleep-SUD interaction. Given that peripheral inflammation has been mechanistically linked to both substance craving and sleep disruption, incorporating an objective, cost-effective inflammatory biomarker could complement subjective assessments, rather than replacing established questionnaires. Thus, the present study aims to elucidate the cross-sectional relationship among SUD, SIRI, and sleep disturbance, specifically testing whether SIRI levels correlate with sleep severity in this population, which will aid in the management of SUD-related sleep disturbance by offering valuable clinical references and insights.

## Methods

### Study population

The NHANES, a large-scale cross-sectional study that utilizes a scientific sampling design to screen representative American population for comprehensive health examinations and interviews, was employed and analyzed (Ahluwalia et al., 2016). This survey protocol has received ethical approval, and all data are publicly available. In this study, data from three consecutive cycles (2013–2014, 2015–2016, and 2017–2018) were used, along with available substance use information (Hinson-Enslin et al., 2022). Participants’ self-reported drug use questionnaires identified specific lifetime substance use patterns, and only two selections were recorded (Guarana et al., 2021): cannabis use with/without other illicit drugs (Verdejo-Garcia and Albein-Urios, 2021); cocaine, heroin, and/or methamphetamine. SUD is diagnosed using the fifth edition of Diagnostic and Statistical Manual of Mental Disorders (DSM-V). Initially, 6,452 participants were included, but the analysis was restricted to 2,533 participants, including SUD group and healthy control cohort 1 (HC1), based on the following exclusion criteria (Guarana et al., 2021): age < 20 years or current pregnancy (Verdejo-Garcia and Albein-Urios, 2021); missing data on education level, marital status, body mass index (BMI), or household income (Adan et al., 2024); smoking or drinking in the past year (Bolling et al., 2025); history of diabetes or hypertension, cardiovascular diseases, prescription drug intake, cancer, or malignancy.

To further validate the results derived from NHANES datasets, between January and December 2024, without human immunodeficiency virus and other infectious diseases, OUD subjects (n = 44), who have used opioids for approximately 18 years, were recruited from Changsha, Hunan Province, China. The primary route of opioid administration in these OUD subjects was hot smoking. This route was determined by self-report during structured clinical interviews. They tested positive for heroin in urine screenings and met the diagnostic criteria for OUD as defined by DSM-V. Age-, education-, gender-, BMI, smoking, and drinking-matched healthy control cohort 2 (HC2) (N = 44) were also voluntarily recruited. All participants were right-handed and had Han Chinese ancestry. No previous diagnoses of head trauma, structural brain disease (e.g., hemorrhage), or seizures, as well as use of antidepressant drugs or anti-inflammatory treatment, were reported in both the OUD group and HC2. On the same day, each subject signed written consent forms and took part in a questionnaire and routine blood examination. The Ethics Committee of the Second Xiangya Hospital in the Central South University approved the research protocol. The workflow of this study is displayed in Figure 1.

**FIGURE 1:**
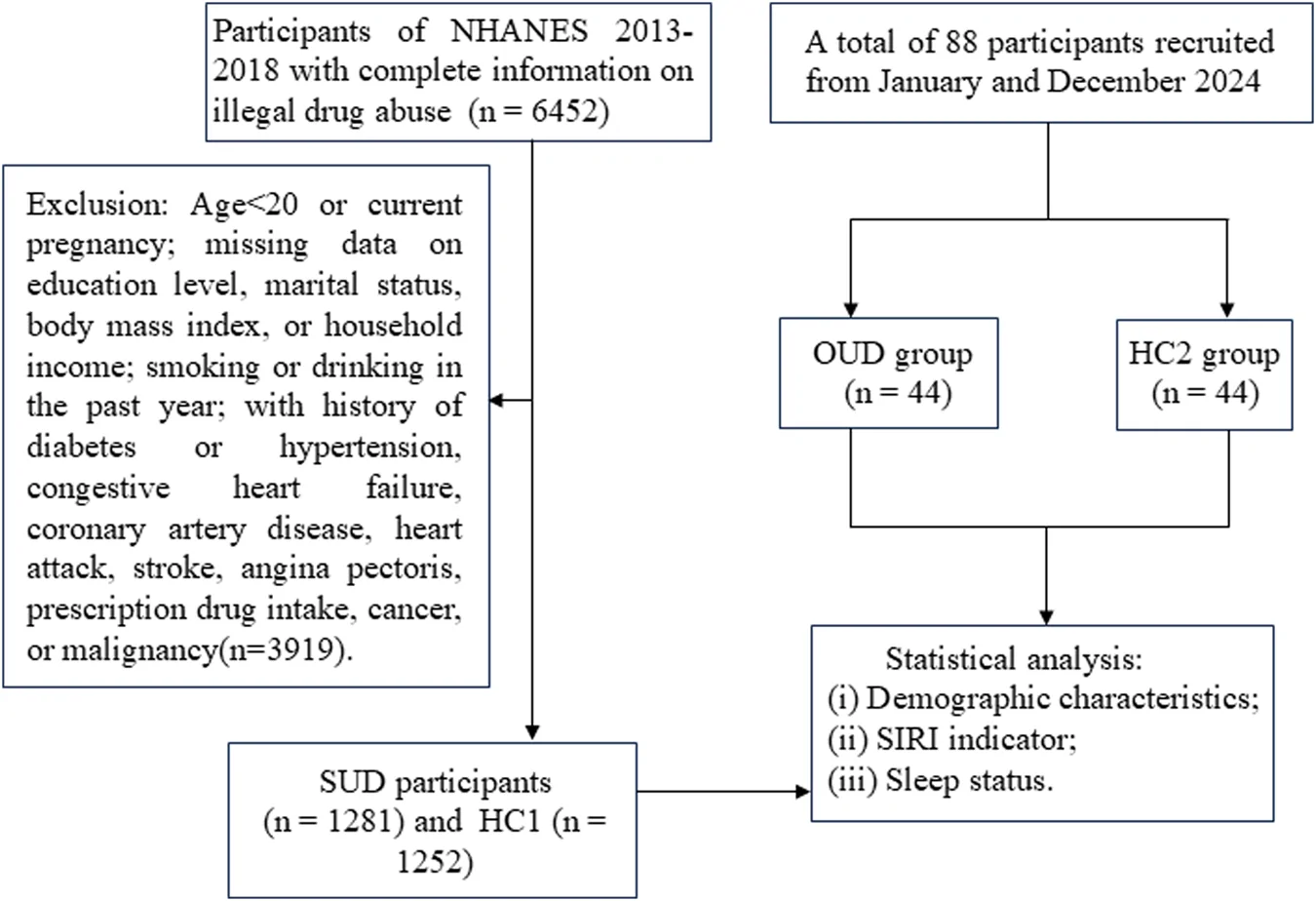
The selection flowchart of clinical and NHANES datasets. HC1, healthy control cohort 1; HC2, healthy control cohort 2; OUD, opioid use disorder; SUD, substance use disorder; SIRI, systemic inflammatory response index.

### Haematological variables

Lymphocyte, neutrophil, platelet, and monocyte counts were measured using automated hematology analysis devices. SIRI is calculated by the following formula (Wang et al., 2022; Wang et al., 2023).

## SIRI = (monocyte count* neutrophil count)/lymphocyte count

### Sleep assessment

The sleep status of participants from NHANES was identified by asking “ever told a doctor or other health professional that you have trouble sleeping”. The answer is “yes” or “no”. The sleep status of OUD and HC2 groups was quantified by PSQI questionnaires (Sancho-Domingo et al., 2021). Sleep quality is assessed using the PSQI in its Chinese version, which consists of 18 items grouped into seven domains, yielding a global score ranging from 0 to 21, where higher scores indicate poorer sleep quality.

### Statistical analysis

IBM SPSS Statistics 26.0 and R 4.5.1 were used to examine statistical analyses in both datasets, with a significance threshold of p < 0.05. For continuous data, normal distributed data manifested by mean ± standard deviation were tested by two-sample t-tests; skewed data manifested by median (25th–75th percentile), were tested using Mann-Whitney tests. Categorical variables were tested by R × C chi-square tests. Following NCHS analysis guidelines, sample weights were applied to all estimates to guarantee better generalization of the non-institutionalized civilian population in the United States In SUD group of NHANES dataset, weighted logistic regression analysis was applied to calculate the odds ratio (OR) and 95% confidence interval (CI) for the relationships between SIRI and sleep disturbance, adjusting for confounders; then, mediation analysis of all included participants from NHANES was performed to reveal association among SUD status, SIRI, and sleep disturbance by taking age, gender, education, race, marital status, annual family income, and BMI as covariates. In OUD group, the association between SIRI and PSQI was conducted via partial correlation analysis by taking age, gender, BMI, education, Fagerstrom Test for Nicotine Dependence, Alcohol Use Disorders Identification Test, and marital status as covariates.

### Machine learning models

As for NHANES, feature selection via the Boruta algorithm was conducted to retain only variables carrying genuine predictive information. Prior to modeling, missing values were handled as follows: continuous variables were imputed with the median and categorical variables with the mode, and variables with excessive missingness (≥ 10%) were excluded from the analysis. Categorical features were transformed using one-hot encoding, and continuous features were standardized (z-score transformation) to ensure scale comparability across algorithms; all preprocessing parameters were estimated on the training set only and then applied to the test set to avoid data leakage. The dataset was randomly divided into training and test sets with a 70%/30% allocation, stratified by the outcome to preserve the prevalence of sleep disturbance in both subsets. The training set, which comprised 70% of the data, was used for hyperparameter tuning and model selection. The model was trained using a 10-fold cross-validation approach. The training set was first partitioned into 10 folds. For each iteration, the model was trained on 9 subsets and validated on the single held-out fold. The overall performance metric for the model was derived by averaging the accuracy from all 10 validation folds. For each algorithm, hyperparameters were optimized within pre-specified candidate ranges using grid search (or randomized search when the parameter space was large), and the combination yielding the highest mean cross-validated area under the ROC curve was selected to refit the model on the entire training set. The model’s generalizability was then ultimately assessed on an independent test set comprising 30% of the original dataset. The optimal model for each algorithm was selected based on the highest area under the ROC. To account for potential class imbalance between participants with and without sleep disturbance, class weights inversely proportional to class frequencies were assigned during training for algorithms supporting sample weighting, thereby ensuring balanced contributions of both classes to model fitting; no resampling was applied to the test set. A set of 11 ML models was adopted for training and testing, including Lasso, CatBoost, Gradient Boosting Tree (GBM), Support Vector Machines (SVM), Decision Tree (DT), LightGBM (LGB), Neural Network (NN), Naive Bayes (NB), XGBoost, Random Forest (RF), and Logistic Regression (LR).

### Selection of candidate variables and predictors

Receiver operating characteristic (ROC), calibration curve, and decision curve defined model performance, with the best-performing model selected for risk prediction of sleep disturbance. Discrimination was quantified by the area under the ROC curve (AUC); calibration, which reflects the agreement between predicted probabilities and observed event frequencies, was evaluated using calibration plots; and clinical utility was assessed by decision curve analysis (DCA) based on the net benefit across a range of threshold probabilities. The model with the highest test-set AUC, satisfactory calibration, and the largest net benefit was designated as the best-performing model for risk prediction of sleep disturbance. To enhance local (individual prediction) and global (dataset-wide) interpretability, Shapley Additive exPlanations (SHAP) analysis was employed to recognize dominant predictors by quantifying the contribution of each feature to model predictions based on game-theoretic Shapley values (Nohara et al., 2022). A positive SHAP value demonstrates a feature increased predicted risk, while a negative value suggests a decrease in risk. The magnitude of the SHAP value reflects the feature’s importance.

## Result

### Clinical characteristics

Differences in clinical features between SUD and HC1 groups were illustrated in Supplementary Table S1. In Table 1, within SUD group, compared to individuals without sleep disturbance, those with sleep disturbance had a higher age, BMI, SIRI, a greater proportion of females (44.5%) and some college or AA degree (43.1%), as well as a larger percentage of widowed (2.3%), divorced (15.6%), and separated individuals (5.7%). Moreover, their annual family income was lower than that of the SUD group without sleep disturbance.

**TABLE 1:** Demographic and clinic characteristics of SUD group stratified by sleep disturbance from NHANES.

Characteristics	Sleep disturbance		Z/χ^2^	p-value
	Yes (n = 436)	No (n = 816)	​	​
Age (years)	43 (33, 52.75)	38 (30, 50)	Z = 4.270	<0.001^*^
Gender				
Male	242 (55.5%)	552 (67.6%)	χ^2^ = 18.060	<0.001^*^
Female	194 (44.5%)	264 (32.4%)		
BMI (kg/m^2^)	31.05 (26.90, 37.03)	28.91 (25.40, 33.17)	Z = 5.457	<0.001^*^
Annual family income	7 (5, 13.75)	8 (6, 14)	Z = −3.354	0.001^*^
Education				
Less than 9th grade	16 (3.7%)	45 (5.5%)	χ^2^ = 11.807	0.019^*^
9–12th grade	52 (11.9%)	124 (15.2%)		
High school graduate	108 (24.8%)	234 (28.7%)		
Some college or AA degree	188 (43.1%)	279 (34.2%)		
College graduate or above	72 (16.5%)	134 (16.4%)		
Marital status				
Married	143 (32.8%)	339 (41.5%)	χ^2^ = 17.155	0.004^*^
Widowed	10 (2.3%)	9 (1.1%)		
Divorced	68 (15.6%)	85 (10.4%)		
Separated	25 (5.7%)	31 (3.8%)		
Never married	117 (26.8%)	224 (27.5%)		
Living with partner	73 (16.7%)	128 (15.7%)		
Race				
Mexican American	46 (10.6%)	166 (20.3%)	χ^2^ = 45.609	<0.001^*^
Other hispanic	40 (9.2%)	65 (8.0%)		
Non-hispanic white	245 (56.2%)	375 (46.0%)		
Non-hispanic black	61 (14.0%)	113 (13.8%)		
Non-hispanic Asian	7 (1.6%)	58 (7.1%)		
Other race	37 (8.5%)	39 (4.8%)		
WBC (×10^9^/L)	7.6 (6.4, 9.3)	7.1 (6, 8.5)	Z = 5.048	<0.001^*^
MONO (×10^9^/L)	0.6 (0.5, 0.7)	0.6 (0.5, 0.7)	Z = 4.314	0.140
NEUT (×10^9^/L)	4.55 (3.6, 5.7)	4 (3.1, 5)	Z = 5.915	0.023^*^
LYMPH (×10^9^/L)	2.2 (1.8, 2.8)	2.2 (1.8, 2.7)	Z = 0.085	0.933
SIRI	1.217 (0.857, 1.835)	1.043 (0.720, 1.400)	Z = 6.389	<0.001^*^

^*^ p < 0.05. BMI, body mass index; LYMPH#, lymphocyte absolute count; MONO#, monocyte absolute count; NEUT#, neutrophil absolute count; WBC, white blood cell; SUD, substance use disorder; SIRI, systemic inflammatory response index.

The clinical characteristics of OUD group and HC2 are summarized in Table 2 and Supplementary Table S2. As exhibited in Table 2, there was no statistically significant difference between OUD group and HC2 in demographic characteristics except for marital status (p < 0.001). OUD group showed higher PSQI score and SIRI than HC2. Comparison of hematological indicators between OUD group and HC2 was shown in Supplementary Table S2.

**TABLE 2:** Demographic characteristics between OUD group and HC2.

Characteristics	OUD (n = 44)	HC2 (n = 44)	t/Z/χ^2^	p-value
Age (years)	53.50 (45.25, 58.75)	53.5 (47, 58.75)	Z = −0.109	0.914
Gender (male/female)	30/14	33/11	χ^2^ = 0.503	0.478
BMI (kg/m^2^)	22.72 (19.66, 25.30)	23.91 (22.20, 26.02)	Z = −1.828	0.068
Education (year)	12 (9, 12)	9 (9, 12)	Z = −1.380	0.168
FTND (score)	4 (2.25, 5.75)	6 (0, 8)	Z = −0.819	0.413
AUDIT (score)	0 (0, 0)	0 (0, 0)	Z = −0.940	0.347
Matrimony (yes/no)	24/20	42/2	χ^2^ = 19.636	0.000^*^
PSQI	11 (5, 13.75)	4.5 (2, 7.75)	Z = −4.442	0.000^*^
SIRI	0.93 ± 0.51	0.74 ± 0.36	t = 2.039	0.045^*^
Gastrointestinal symptoms (yes/no)	0/44	0/44	/	
Usage of antidepressant drugs (yes/no)	0/44	0/44		
Anti-inflammatory treatment (yes/no)	0/44	0/44		
Opioid condition				
Intake method (hot smoking/others)	44/0			
Age of first opioid use (year)	24 (20.25, 28.75)			
Duration of opioid use (year)	20 (14.25, 25)			
Age of regular opioid use (year)	25 (21, 29)			
Duration of regular opioid use (year)	18 (13.25, 23.75)			
Times of opioid relapse (times)	2 (1, 3.75)			

^*^ p < 0.05. Normal distributed data are in the form of mean ± SD (standard deviation). Skewed data are exhibited by median (25% confidence interval∼75% confidence interval). AUDIT, alcohol use disorders identification test; BMI, body mass index; FTND, fagerstrom test for nicotine dependence; HC2, healthy control-cohort2; OUD, opioid use disorder; PSQI, pittsburgh sleep quality index; SIRI, systemic inflammatory response index.

### Association between sleep disturbance and SIRI in SUD participants

Table 3 presents the relationship between SIRI and sleep disturbance in SUD population from NHANES dataset. Three models were used in this study. After adjusting age, gender, education, race, marital status, annual family income, and BMI, Model 3 showed that compared with SIRI < 0.766, OR with 95% CI was 1.15 (1.07–1.24, p = 0.003) among SIRI 0.776–1.125; OR with 95% CI was 1.29 (1.19–1.39, p = 1.39 × 10^−6^) among SIRI 1.125–1.622; OR with 95% CI was 2.00 (1.85–2.17, p = 5.44 × 10^−15^) among SIRI > 1.622. For the self-collected datasets, we also found that the SIRI was positively related to PSQI (r = 0.327, p = 0.037) in OUD group (Figure 2). Collectively, both results of public and self-collected datasets indicated that SIRI was positively correlated with sleep disturbance.

**TABLE 3:** The associations of SIRI with sleep disturbance in substance use disorder participants from NHANES datasets.

SIRI	Model 1		Model 2		Model 3	
	OR (95%CI)	p-value	OR (95%CI)	p-value	OR (95%CI)	p-value
Q1 (< 0.766)	Ref		Ref		Ref	
Q2 (0.766–1.125)	1.15 (1.06–1.26)	0.003	1.15 (1.06–1.25)	0.003	1.15 (1.07–1.24)	0.003
Q3 (1.125–1.622)	1.33 (1.22–1.45)	0.000^*^	1.31 (1.20–1.43)	0.000^*^	1.29 (1.19–1.39)	0.000^*^
Q4 (> 1.622)	2.09 (1.92–2.27)	0.000^*^	2.06 (1.90–2.24)	0.000^*^	2.00 (1.85–2.17)	0.000^*^

^*^ p < 0.001. Model 1, did not adjust for any confounders; Model 2, adjusted for age, gender, education, and race; Model 3, adjusted for age, gender, education, race, marital status, annual family income, and BMI. BMI, body mass index; CI, confidence interval; OR, odds ratio; SIRI, systemic inflammatory response index.

**FIGURE 2:**
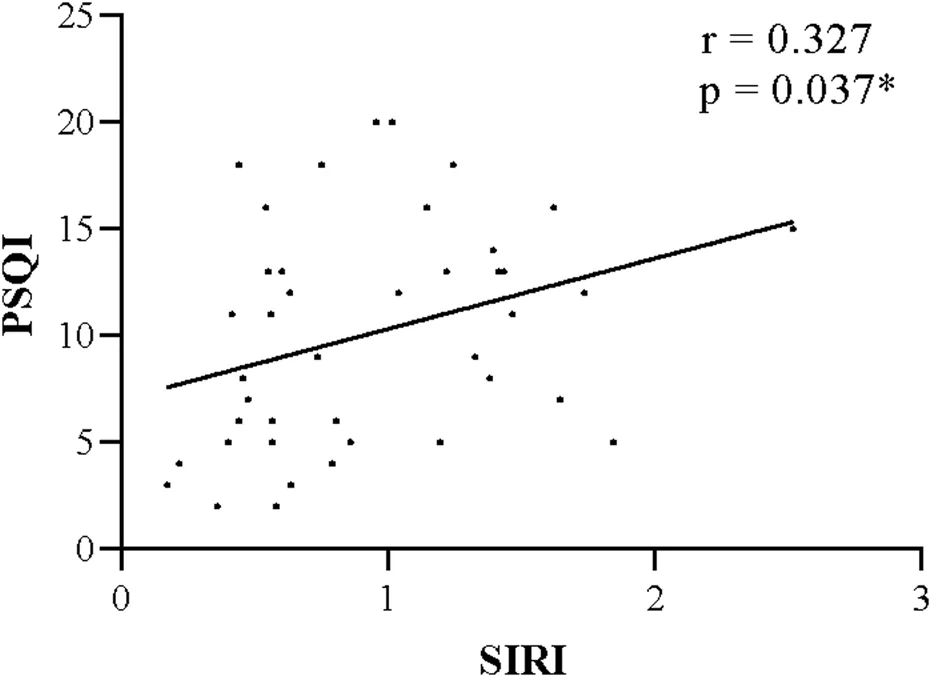
Association of PSQI score and SIRI in the OUD group from clinical dataset. PSQI, Pittsburgh sleep quality index; SIRI, systemic inflammatory response index.

### Mediation analysis: SIRI as a potential mediator linking SUD to sleep disturbance

To further investigate the relationship among SUD status, SIRI, and sleep disturbance, mediation analysis was conducted among exposure [SUD status (Binary categorical variable; SUD vs. HC1)], mediator [SIRI (Continuous variable)], and outcome [sleep disturbance (Binary categorical variable; yes vs. no)] in all subjects from NHANES dataset. The total effect, average direct effects (direct effect), and average causal mediation effects (indirect effect) of “SUD - SIRI - sleep disturbance” mediation pathway were 0.168 (95% CI: [0.120, 0.216], p < 2.2 × 10^−16^), 0.128 (95% CI: [0.080, 0.173], p < 2.2 × 10^−16^), and 0.040 (95% CI: [0.026, 0.056], p < 2.2 × 10^−16^). The proportion of mediated effect was 0.239 (95% CI: [0.153, 0.351], p < 2.2 × 10^−16^).

### Performance of multiple machine learning models

Utilizing features with p < 0.05 (Figure 3), we performed extensive model training and evaluation. In the training set, the AUC for all models ranged from 0.500 to 0.999, with the CatBoost and RF model achieving the highest performance (AUC: 1, 0.996, respectively) (Figure 4A), and a certain degree of overfitting may be present. Calibration curves for multiple ML models in the training set (Figure 4B) revealed that the RF model manifested excellent calibration properties. DCA of the training set (Figure 4C) indicated that both CatBoost and RF models yielded larger net benefit areas. For the test set, the area under the ROC ranged from 0.500 to 0.880, with CatBoost and RF achieving the highest performance (AUC: 0.880, 0.846, respectively) (Figure 4D). The test set calibration curves (Figure 4E) and DCA (Figure 4F) respectively demonstrated optimal performance and the most net benefit areas for the CatBoost model. On the training set, the AUC for 11 ML algorithms were: Lasso (0.694), CatBoost (0.999), GBM (0.774), SVM (0.802), DT (0.564), LGB (0.500), NN (0.694), NB (0.725), XGBoost (0.776), RF (0.996), and LR (0.694). On the test set, the AUC values were as follows: Lasso (0.619), CatBoost (0.880), GBM (0.5), SVM (0.656), DT (0.534), LGB (0.5), NN (0.620), NB (0.625), XGBoost (0.688), RF (0.846), and LR (0.619). Integrating the findings of ROC, calibration curves, and DCA, we selected both CatBoost and RF as top-performing algorithms, which were subsequently used for feature importance analysis and selection.

**FIGURE 3:**
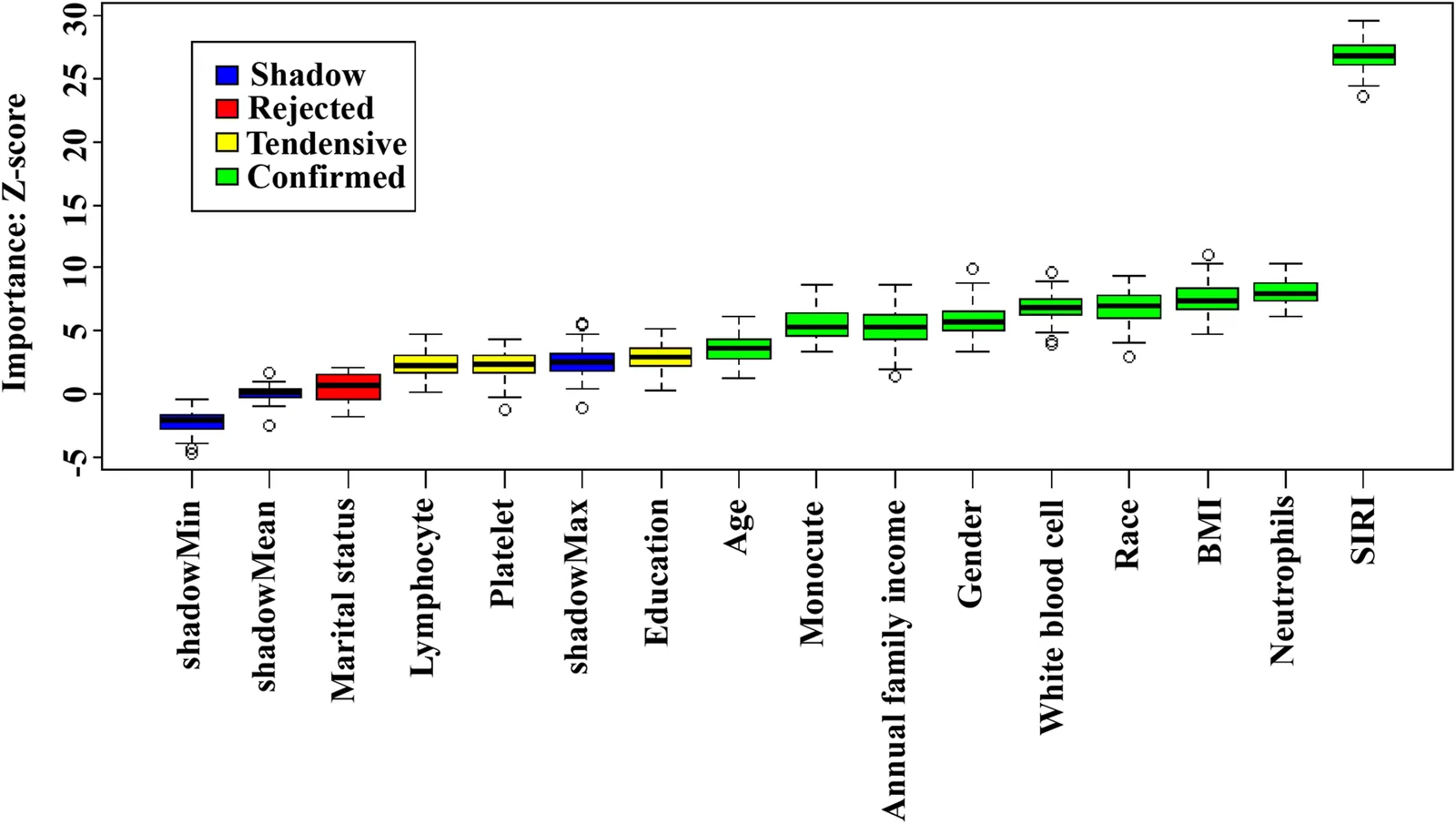
Feature importance ranking of clinical and laboratory variables in predicting sleep disturbance. The Z-score represents the standardized importance of each variable in the model. BMI, body mass index; SIRI, systemic inflammatory response index.

**FIGURE 4:**
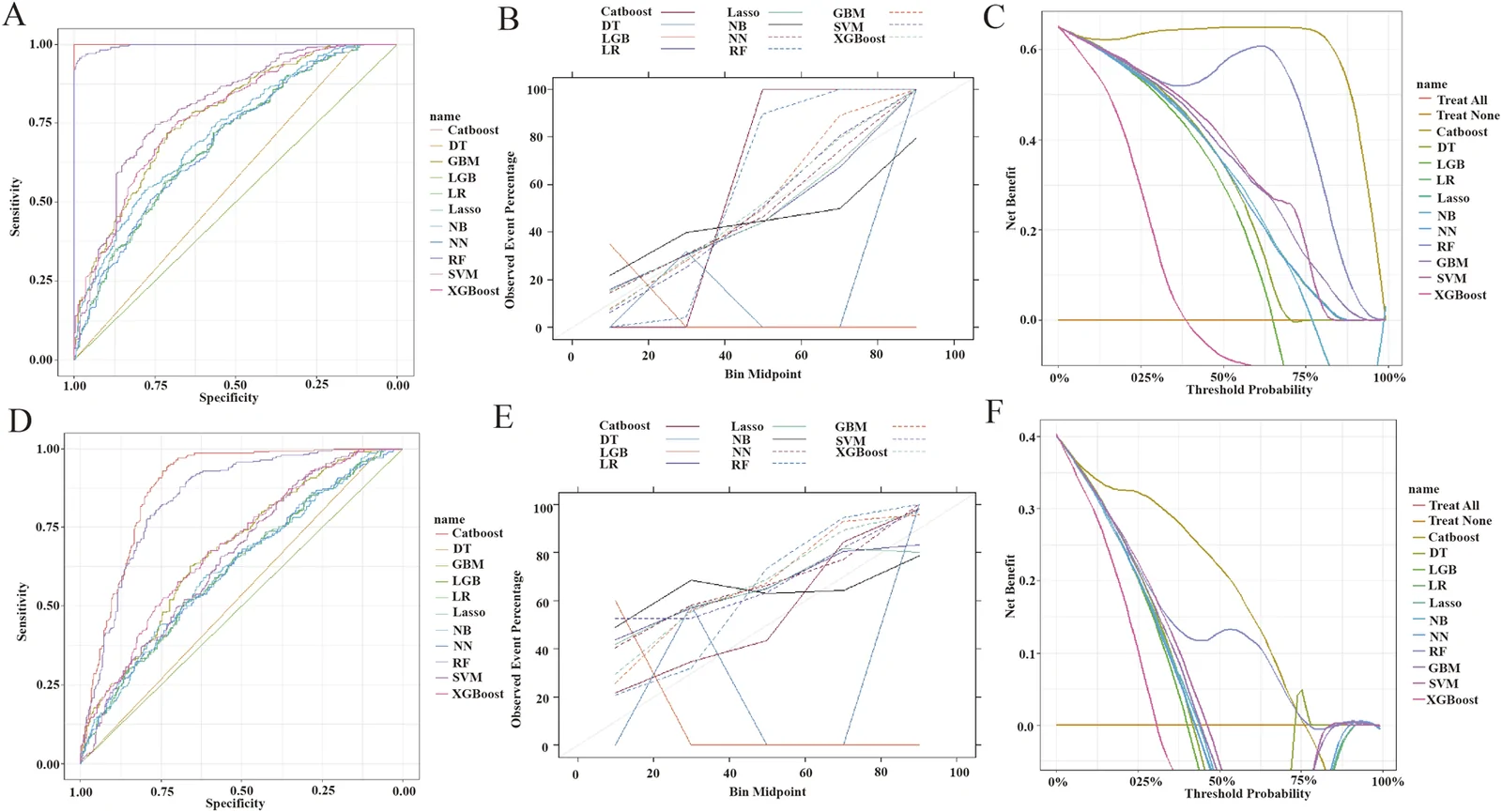
Performance evaluation curves for 11 machine learning models in identifying sleep disturbance within substance users from NHANES. **(A)** ROC curve analysis in the training data. **(B)** Calibration curve analysis in the training data. **(C)** Decision curve analysis in the training data. **(D)** ROC curve analysis in the test data. **(E)** Calibration curve analysis in the test data. **(F)** Decision curve analysis in the test data. DT, Decision Tree; GBM, Gradient Boosting Tree; LGB, LightGBM; LR, Logistic Regression; NB, Naive Bayes; NN, Neural Network; SVM, Support Vector Machines; RF, Random Forest. Supplementary Figure S1. Machine learning algorithms for identifying important features. **(A)** Bar plot of feature importance based on CatBoost. **(B)** Evaluation of SHAP values across all features for an individual instance based on Catboost. **(C)** Beeswarm plot of feature effects based on Catboost-SHAP. **(D)** Bar plot of feature importance based on RF. **(E)** Evaluation of SHAP values across all features for an individual instance based on RF. **(F)** Beeswarm plot of feature effects based on RF-SHAP. BMI, body mass index; RF, Random Forest; SHAP, Shapley Additive exPlanations; SIRI, systemic inflammatory response index.

### Variable importance and variable interpretation

The key features recognized by CatBoost were depicted in Supplementary Figure S1A, and the top 5 features were SIRI, race, BMI, age, and gender. As exhibited in Supplementary Figure S1B, in a specific sample, the expected value f(x) is 1.87, higher than E (f(x)) (0), indicating an elevated level of sleep disturbance in this individual. SHAP interpretation attributes this higher prediction primarily to variables such as BMI, race, and SIRI. Among the 9 key features, SIRI had the most significant impact, indicating that greater SIRI correlated with a higher risk of sleep disturbance (Supplementary Figure S1C). The important features identified by RF are shown in Supplementary Figure S1D, and the top 5 features are SIRI, neutrophils, BMI, monocytes, and white blood cells. In Supplementary Figure S1E, the expected value f(x) for a specific sample is 1767, higher than E (f(x)) (0), suggesting an above-average sleep disturbance level for this individual. This is mainly affected by SIRI and neutrophils. In Supplementary Figure S1F, SIRI serves as the most significant index influencing sleep disturbance.

## Discussion

Through NHANES and clinical databases, this work delved into the roles of SIRI in SUD-related sleep disturbance. Firstly, in NHANES dataset, multivariate logistic regression analysis, mediation analysis, and multiple ML models were used to investigate the “SUD-SIRI-sleep disturbance” relationship and the importance of SIRI among top diagnostic features. The proportion of mediated effect was 0.239 (95% CI: [0.153, 0.351], p < 2.2 × 10^−16^), indicating that approximately 23.9% of the total association between SUD and sleep disturbance was mediated through SIRI. Given the highly multifactorial etiology of sleep disturbance in patients with SUD, the finding that single SIRI accounts for nearly one-quarter of the total effect is substantial and noteworthy, highlighting SIRI as a critical partial mediator. The remaining 76.1% of the association was either direct or mediated through other unmeasured pathways. Next, using clinical sources, we further discovered SIRI abnormality and a positive “SIRI-PSQI” correlation in OUD group. SIRI may partially mediate the association between SUD and sleep disturbance. During classification of sleep status within SUD participants, SIRI had the highest importance score. We proposed that clinically, SIRI may serve as a potential mediating biomarker linking substance use to sleep disturbance, enabling early risk stratification and offering potential evidence for anti-inflammatory interventions to improve sleep quality in affected individuals.

Substance use leads to a peripheral pro-inflammatory response represented by greater SIRI. For example, exogenous opioids exerted immunomodulatory effects via toll-like receptor-4, which is notably expressed on myeloid cells (e.g., neutrophils, monocytes) (Grace et al., 2015). This indicated that OUD-induced aberrations in peripheral pro-inflammatory cytokines and immune cells might be monitoring indicators or candidate targets for the severity of neuropsychological symptoms (Butelman et al., 2023). Opioids facilitated the peripheral expansion of fragile-like regulatory T cells, which infiltrated into accumbens through disrupted blood-brain barrier (BBB), and then mediated synaptic instability of NAc via Interferon-γ, contributing to abnormal neuropsychiatric symptoms (Zhu et al., 2023). Interestingly, minocycline might have a neuroprotective role in BBB integrity through the suppression of matrix metalloproteases, which could also block T-cell infiltration (Romero-Miguel et al., 2021). Consistent with previous research, this study indicated an elevated systemic inflammation in drug misusers. Additionally, although our limited sample size precluded a subgroup analysis specifically focusing on constipated patients, it is still important to note that systemic pro-inflammatory response may also originate from long-term intestinal dysfunction. Constipation observed in patients with OUD represents one of the most prevalent and clinically significant manifestations of opioid-induced bowel dysfunction. Opioids bind to intestinal μ-opioid receptors, resulting in reduced intestinal peristalsis and constipation (Becker et al., 2026). Long-standing opioid-induced constipation may modulate circulating neutrophils and pro-inflammatory cytokines through mechanisms including gut dysbiosis, impaired intestinal barrier integrity, and LPS activation (Bettinger and Friedman, 2024). Nevertheless, this is often intertwined with multiple factors, such as the intrinsic immunomodulatory effects of opioids, intestinal function status, co-infection, and alterations of immune microenvironment. Changes in circulating innate immune cells (particularly neutrophils) among patients with OUD thus constitute a highly dynamic, pathophysiologically complex process. Accordingly, comprehensive evaluation and consideration of the patient’s overall clinical context are warranted when employing this biomarker.

Higher SIRI is associated with poorer sleep in patients with SUD. Through active mechanisms and cellular pathways, inflammatory signals can be conveyed to the central nervous system via the actions of humoral mediators, direct neural innervation, and blood-brain barrier transport (Huang et al., 2021). The activities of key neurotransmitter systems that regulate behaviour (such as serotonin, dopamine, and noradrenaline) are attenuated and can result in alterations in sleep patterns (Yeh et al., 2023). It was demonstrated that pharmacological administration of IL-6 suppressed early-night slow-wave and non-rapid eye movement sleep and reduced rapid-eye movement sleep in humans (Irwin, 2019). In turn, sleep disturbance can activate pro-inflammatory mechanisms (Yeh et al., 2023). The imbalance of Th1/Th2 and M1/M2 activity was one of the pathophysiological responses of SUD (11). Monocytes and neutrophils are major cells participating in innate immunity regulation and can generate multiple pro-inflammatory factors (e.g., TNF-α, IL-6) (Arivazhagan et al., 2020), which cause pathological damage related to psychiatric disorders (e.g., sleep disorder, SUD) (Piber et al., 2022; Miller et al., 2018). Thus, it suggests that SIRI may function as an accessible indicator for evaluating the “SUD-sleep disturbance” axis, thereby informing the development of prognostic management and even targeted interventions. We propose a risk-stratification framework rather than a diagnostic threshold: patients with SUD and persistently elevated SIRI may represent a pro-inflammatory endotype warranting closer monitoring for sleep deterioration, treatment resistance, or relapse vulnerability. We emphasize that SIRI should be used as a supplementary indicator to be integrated with subjective sleep assessments (e.g., PSQI, ISI) and clinical judgment, rather than as a standalone diagnostic or prognostic tool.

This work has several merits. Firstly, the analyses are conducted on an extensive sample from the NHANES, which provides a high degree of representativeness and reliability for this cross-sectional study. Secondly, the study adopted a complex multi-stage probability sampling design and extensive covariate adjustment, enhancing the precision of statistical inference and credible findings in the surveyed population. Thirdly, an extra clinical dataset further confirmed the relationships between SIRI and sleep disturbance. However, there are several limitations. Firstly, although the mediation analysis inferred the “SUD-SIRI-sleep disturbance” axis in this cross-sectional study, the causal relationships among them need to be further confirmed by longitudinal follow-up or experimental validation. Secondly, the sleep assessments of both datasets are self-reported with inherent reporting and recall biases. Notably, misclassification from the single-item NHANES measure would likely bias effect estimates towards the null, yielding more conservative results. Therefore, the significant associations observed in our study may represent underestimates of the true effects, further supporting the robustness of our main findings. NHANES adopts sleep assessments that differ in evaluation dimensions from the PSQI used in the clinical cohort, potentially leading to bias when comparing results across studies. Thirdly, the sample size of our self-collected dataset, which was confined to OUD patients under relatively “pure” conditions (i.e., excluding major psychiatric comorbidities, antidepression medication use, and anti-inflammatory treatment), is modest. This restricts the generalizability of our findings to the broader, more heterogeneous OUD population encountered in real-world clinical practice. The findings deserve to be further validated in other types of SUD (e.g., opioids, cannabis, methamphetamine, cocaine) to assess the generalization ability of our results. Finally, there is a lack of detailed records of multiple drug use information in NHANES samples included in this study, which hindered further subgroup analysis. For example, the impact of different inhalation methods on peripheral immune indicators may vary, especially for intravenous injection that significantly increases peripheral neutrophils in the short term. The NHANES dataset may contain some intravenous injection patients, making it difficult to confirm whether it is a long-term effect or a short-term sudden change. Clinical research can only provide evidence. Additionally, the near-perfect training AUCs of the CatBoost and RF models suggested a certain degree of overfitting; although their performance on the independent test set remained the highest among all algorithms, external validation in larger, independent cohorts is warranted to further confirm the generalizability of these models.

In conclusion, this study underscores the critical role of SIRI as a mediator in the relationship between SUD and sleep disturbance, which paves the way to further investigate biological mechanisms underlying their relationship. Therefore, it provides a promising indicator for dynamically monitoring sleep disturbance by targeting inflammation, paving the way for developing more effective therapeutic paradigms to mitigate the psychiatric burden of individuals with SUD and consequently improving overall health outcomes. Future investigation should pay more attention to this relationship, with an emphasis on the potential for personalized treatment approaches and interventions.

## Data Availability

The raw data supporting the conclusions of this article will be made available by the authors, without undue reservation.
