# Supplementary material for: Systemic inflammatory response index: a potential mediator between substance use disorder and sleep disturbance
Source: Front Pharmacol. 2026 Oct 20;17:1964417. doi: 10.3389/fphar.2026.1964417 (PMC13727624; doi:10.3389/fphar.2026.1964417)
Supplement: Supplementary file 1 [file Table1.docx]

**Table S1.** Demographic and clinic characteristics of SU and NSU groups from NHANES.

| Characteristics | SUD (n=1252) | HC1 (n=1281) | Z/χ^2^ | p-value |
| --- | --- | --- | --- | --- |
| Age (years) | 40(31, 51) | 33(27, 40) | Z=15.741 | <0.001^*^ |
| Gender | | | | |
| male | 794(63.4%) | 0(0%) | χ^2^=1183 | <0.001^*^ |
| female | 458(36.6%) | 1281(100%) |  |  |
| BMI (kg/m^2^) | 29.64(25.83, 34.45) | 28.6(24.4, 35.1) | Z=2.790 | 0.005^*^ |
| Annual family income | 8(5, 14) | 8 (6, 14) |  |  |
| Education | | | | |
| Less than 9th grade | 61(4.9%) | 83(6.5%) | χ^2^=100 | <0.001^*^ |
| 9-12th grade | 176(14.1%) | 128(10.0%) |  |  |
| High school graduate | 342(27.3%) | 236(18.4%) |  |  |
| Some college or AA degree | 467(37.3%) | 424(33.1%) |  |  |
| College graduate or above | 206(16.5%) | 410(32.0%) |  |  |
| Marital status | | | | |
| Married | 482(38.5%) | 687(53.6%) | χ^2^=77 | <0.001^*^ |
| Widowed | 19(1.5%) | 10(0.8%) |  |  |
| Divorced | 153(12.2%) | 74(5.8%) |  |  |
| Separated | 56(4.5%) | 35(2.7%) |  |  |
| Never married | 341(27.2%) | 317(24.7%) |  |  |
| Living with partner | 201(16.1%) | 158(12.3%) |  |  |
| Race | | | | |
| Mexican American | 212(16.9%) | 273(21.3%) | χ^2^=248 | <0.001^*^ |
| Other Hispanic | 105(8.4%) | 156(12.2%) |  |  |
| Non-Hispanic White | 620(49.5%) | 319(24.9%) |  |  |
| Non-Hispanic Black | 174(13.9%) | 237(18.5%) |  |  |
| Non-Hispanic Asian | 65(5.2%) | 256(20.0%) |  |  |
| Other Race | 76(6.1%) | 40(3.1%) |  |  |
| WBC (×10^9^/L) | 7.3(6.1, 8.8) | 7.3(6, 8.9) | Z=-0.312 | 0.755 |
| MONO (×10^9^/L) | 0.6(0.5, 0.7) | 0.5(0.4, 0.6) | Z=7.876 | <0.001^*^ |
| NEUT(×10^9^/L) | 4.2(3.3, 5.3) | 4.3(3.3, 5.4) | Z=0.884 | 0.377 |
| LYMPH (×10^9^/L) | 2.2(1.8, 2.7) | 2.2(1.8, 2.7) | Z=-0.567 | 0.570 |
| SIRI | 1.107(0.772, 1.543) | 1(0.694, 1.400) | Z=4.689 | <0.001^*^ |
| Sleep disturbance | | | | |
| Yes | 436(34.8%) | 208(16.2%) | χ^2^=115 | <0.001^*^ |
| No | 816(65.2%) | 1073(83.8%) |  |  |
| Substance use | | | | |
| Cannabis use with/without other illicit drug (cocaine, heroin, and/or methamphetamine) | 1188(94.9%) | | | |
| Cocaine, heroin, and/or methamphetamine | 64(5.1%) | | | |

SUD, substance use disorder; HC1, healthy control-cohort1; LYMPH#, lymphocyte absolute count; MONO#, monocyte absolute count; NEUT#, neutrophil absolute count; WBC, white blood cell; SIRI, systemic inflammatory response index.

**Table S2.** Summary of blood routine examination in OUD group and HC2.

| Routine blood test indicators | OUD (n=44) | HC2 (n=44) | t/Z | p-value |
| --- | --- | --- | --- | --- |
| WBC (×10^9^/L) | 6.31±1.65 | 6.18±1.78 | t=0.352 | 0.726 |
| HGB (g/L) | 140.05±13.13 | 142.09±18.80 | t=-0.592 | 0.556 |
| RBC (×10^12^/L) | 4.59±0.41 | 4.84±0.72 | t=-2.062 | 0.042^*^ |
| PLT (×10^9^/L) | 176.45±58.65 | 227.20±47.89 | t=-4.446 | 0.000^*^ |
| LYMPH (×10^9^/L) | 2.10(1.75,2.59) | 2.04(1.63, 2.61) | Z=-0.204 | 0.839 |
| LYMPH (%) | 34.81±10.23 | 35.05±7.97 | t=-0.120 | 0.905 |
| NEUT(×10^9^/L) | 3.42±1.31 | 3.36±1.14 | t=0.221 | 0.825 |
| NEUT (%) | 53.56±10.64 | 53.97±7.77 | t=-0.207 | 0.836 |
| MONO (×10^9^/L) | 0.52(0.40, 0.61) | 0.43(0.33, 0.60) | Z=-2.001 | 0.045^*^ |
| MONO (%) | 8.30(7.50, 9.90) | 7.40(6.13, 7.98) | Z=-3.138 | 0.002^*^ |
| MCV (fL) | 93.55(90.40, 95.35) | 92.85(89.50, 95.20) | Z=-1.110 | 0.267 |
| MCH (pg) | 30.85(29.70, 31.80) | 30.10(29.40, 30.88) | Z=-1.816 | 0.069 |
| BASO (×10^9^/L) | 0.035±0.015 | 0.039±0.017 | t=-1.214 | 0.228 |
| BASO (%) | 0.57±0.27 | 0.65±0.27 | t=-1.312 | 0.193 |
| EO (×10^9^/L) | 0.11(0.05, 0.20) | 0.16(0.10, 0.22) | Z=-1.701 | 0.089 |
| EO (%) | 1.90(1.10, 2.90) | 2.45(1.63, 4.13) | Z=-1.996 | 0.046^*^ |
| IG (×10^9^/L) | 0.01(0.01, 0.02) | 0.01(0.01, 0.02) | Z=-0.937 | 0.349 |
| IG (%) | 0.20(0.10, 0.30) | 0.20(0.10, 0.30) | Z=-0.805 | 0.421 |
| HCT (%) | 42.75±3.25 | 43.71±5.04 | t=-1.059 | 0.293 |
| RDW-CV (%) | 12.90(12.40, 13.20) | 12.75(12.33, 13.45) | Z=-0.085 | 0.932 |
| RDW-SD | 44.40±3.07 | 44.49±4.08 | t=-0.114 | 0.909 |
| MCHC (g/L) | 327.25±11.93 | 324.34±10.87 | t=1.195 | 0.235 |
| P-LCR (%) | 28.16±7.97 | 29.42±7.13 | t=-0.762 | 0.448 |
| PDW (fL) | 12.27±2.25 | 12.43±1.94 | t=-0.358 | 0.721 |
| MPV (fL) | 10.39±0.95 | 10.62±0.89 | t=-1.116 | 0.268 |
| PCT (%) | 0.19(0.14, 0.22) | 0.24(0.22, 0.28) | Z=-4.877 | 0.000^*^ |

^*^p<0.05. Normal distributed data are in the form of mean ± SD (standard deviation). Skewed data are exhibited by median (25% confidence interval~75% confidence interval). BASO#, basophil absolute count; BASO%, basophil percentage; EO#, eosinophil absolute count; EO%, eosinophil percentage; HC2, healthy control-cohort2; HCT, hematocrit; HGB, hemoglobin; IG#, immature granulocyte count; IG%, immature granulocyte percentage; LYMPH#, lymphocyte absolute count; LYMPH%, lymphocyte percentage; MCH, mean corpuscular hemoglobin; MCHC, mean corpuscular hemoglobin concentration; MCV, mean corpuscular volume; MD, mean diffusivity; MPV, mean platelet volume; MONO#, monocyte absolute count; MONO%, monocyte percentage; NEUT#, neutrophil absolute count; NEUT%, neutrophil percentage; PLT, platelet; PCT, platelet critical volume; PDW, platelet distribution width; P-LCR, platelet large cell ratio; RBC, red blood cell; RD, radial diffusivity; RDW-CV, red cell distribution width-coefficient of variation; RDW-SD, red cell distribution width-standard deviation; WBC, white blood cell.
